# 49-years-old women with unusual presentation of severe hypokalemia mimicking Guillain-Barré Syndrome: A rare case report

**DOI:** 10.1016/j.amsu.2022.104021

**Published:** 2022-06-18

**Authors:** Mohamed Mukhtar Mohamed, Ibrahim Hussein Ali, Mehmet Erdem Ağca, Mohamed Sheikh Hassan, Mohamed Farah Osman, Irshad Ibrahim Ali, Mohamed Farah Yusuf Mohamud

**Affiliations:** Mogadishu Somali Turkey Research and Training Hospital, Mogadishu, Somalia

**Keywords:** Hypokalimia, Guillain-Barré Syndrome, Quadraparesis, Emergency

## Abstract

Acute neuromuscular weakness with associated subsequent developing respiratory failure is common neurological emergency in all emergency departments worldwide. Guillain-Barré Syndrome (GBS) remains the most common cause of acute ascending quadriplegia presents with ascending muscle weakness associated with paresthesia and loss of deep tendon reflexes and usually preceeded by diarrheal illness or upper airway infection. Here we report 49-year-old female presented with rapidly progressing, ascending quadraparesis for 48 hours duration with subsequently complicated by respiratory paralysis due to severe hypokalemia.

## Highlights

• Highlights (For Review).
• Acute neuromuscular weakness with associated respiratory failure is not an uncommon problem to emergency departments all over the world, including Somalia.
• Even though acute hypokalemia induced areflexic weakness is a rare disorder, it is a potentially treatable and reversible cause of acute ascending areflexic muscle weakness.
• From this case report is, every physician and intensivist working in emergency room, especially in resource limited setting whenever they are faced with a patient presenting to ER with acute ascending flaccid quadriparesis without sensory deficit, they should determine serum electrolyte for every such patient to consider severe hypokalemia as possible & treatable differential diagnosis before unnecessary investigations and costly interventions.

## Abbreviations

GBS: Guillain-Barre syndrome
AIDP: Acute inflammatory demylinating polyradiculoneuropathy
AMAN: Acute Motor Axonal Neuropathy
ER: Emergency Room
ICU: Intensive Care Unit

## 1 Introduction

Acute neuromuscular weakness with associated respiratory failure is not an uncommon problem to emergency departments all over the world, including Somalia. The differential diagnosis includes neurological problems, metabolic disorders and infectious disease [1]

**Table 1:** Summarizing of Laboratory results.

Blood investigation	At admission	After 24hr of admission	On Discharge
WBC	6.29	13.02	12
HGB	10.9	9.3	9.1
PLT	463	376	249
Urea	40	45	26
Creatinine	0.96	0.93	0.55
AST	31	26	179
ALT	10	7	84
Potassium	**1.72**	2.77	3.61
Sodium	149	155	145
Magnesium	–	1.73	**2.06**
Calcium	8.8	9.2	7.8
Albium	3.7	3.5	3.2
Glucose	193	145	99
pH	**7.12**	7.26	7.40
Pco2	38.5	35.2	33
CHCO(P)	12.5	15.8	22

GBS is an autoimmune polyradiculoneuropathy in which involves peripheral nerve myelin sheeth and it is secondary to minor infectious illness like acute gastroenteritis, upper respiratory infection, and some vaccinations [2].

Guillain-Barré Syndrome (GBS) remains the most common cause of acute ascending quadriplegia presents with ascending muscle weakness associated with paresthesia and loss of deep tendon reflexes [3].

Since any of the above may have symptoms similar to those found in GBS, a rapid certain diagnosis may be difficult. Even though acute hypokalemia induced areflexic weakness is a rare disorder, it is a potentially treatable and reversible cause of acute ascending areflexic muscle weakness [4].

Here we report a case of severe hypokalemia presented with rapidly progressing, ascending weakness of all four extremities and increasing difficulty breathing mimicking Guillain-Barré Syndrome.

## 2 Case report

A 49 year-old female was brought to emergency Department of Mogadishu Somali turkey research and training hospital by her family with rapidly progressing, ascending weakness of all four extremities for 48 hours duration, During that time she was also having increasing difficulty breathing.

There was no history of similar condition in past or in her family; also no history of trauma, chronic Disease or intake of any drugs prior to this illness, also there was no any history of loss of consciousness, abnormal body movement, urinary retention or incontinence. The patient denies any history of recent vaccination, flu symptoms or exposure to barium or any other drugs. Her social history denies khat chewing & drinking any alcohol.

On examination, the patient was fully conscious and oriented but in acute respiratory distress wearing a face mask with high oxygen flow supplementation. With blood pressure 140/90 mmHg, pulse rate 98 beats/minute, respiratory rate 40breaths/minute and oxygen saturation 92% with oxygen by oxygen face mask. On respiratory system examination there was visible use of use of accessory muscles and Chest auscultation was clear. Neurological examination revealed hypotonia in all four limbs (lower extremity > upper extremity) with muscle power 1/5 in lower limbs and 3/5 in upper limbs with absent deep tendon reflexes and bilateral equivocal plantar responses. There was no sensory deficit and cranial nerve abnormality. Pupils were normal in size and reaction to light.

Her abdomen was distended with absent bowel sounds but no guarding, tenderness or rigidity. A provisional diagnosis of GBS was made and supportive management started. Within few hours, the patient started further developing signs of respiratory distress & we were intubated with ventilatory support. She was kept in the emergency department while waiting the intensive care unit (ICU) transfer also We ordered Analyses of blood samples sent for arterial blood gases, biochemistry including electrolytes (sodium potassium calcium),blood glucose, renal function & liver function tests and complete blood count, coagulation and Elisa profiles; which revealed low potassium (K+ = 1.72 mEq/L in biochemistry) & (K+ = 1.38 mEq/L in arterial blood gases),her blood gases revealed metabolic acidosis and hypokalemia pH 7.12, HCO3- 12.5 mmol/L, BE -15.8 mmol/L, pCO2 38.5 mmHg), normal sodium 149mEq/l and calcium 8.8mg/dl, her blood sugar, renal function, liver function and coagulation profiles were within normal range. her hemogram revealed moderate anemia with normal other parameters, the Elisa screenings tested negative to hepatitis B, C & HIV (Table 1).

**Fig. 1:**
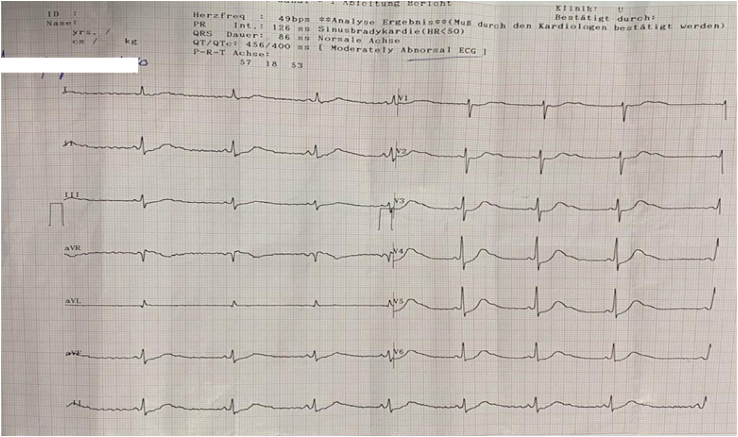
The EKG of patient showed ST depression and T-wave inversion (Hypokalemia).

ECG showed ST depression and T-wave inversion (Fig. 1).the patient is shifted to intensive care unit (ICU) and the diagnosis of hypokalemia induced paralysis impending type 2 respiratory failures is considered and management of hypokalemia with correction of metabolic acidosis started.#

Following the ICU admission the patient was given parenteral potassium chloride by using **K**_**deficit**_ **(in mmol) = (K**_**normal**_ _**lower**_ _**limit**_ **− K**_**measured**_**) × kg body weight × 0.4 plus daily required potassium which 1 mmol per kg.** In this patient, the calculated deficit would be [(3.5–1.72)*50kg*0.4 = 35.6mmol] + (1mmol*50kg = 50mmol) = 86 mmol/24hour. First we give 43 milliequivalents in 500 mL normal saline over 4 hours and the remaining 43 milliequalivant of potassium chloride in 500ml of normal saline was given as maintenance. Furthermore, 80mg in 8 ampoules of sodium bicarbonate in 500 normal saline was given as infusion for the correction metabolic acidosis.

In the next morning Serum potassium,arterial blood gases was repeated and magnesium levels ordered which revealed hypokalemia (2.77 mEq/L), hypomagnesia (1.73mg/dl) normal range (1.87–2.5mg/dll) and improvement in metabolic acidosis (pH 7.26, HCO3- 15.8 mmol/L, BE -10.4 mmol/L, pCO2 35.2 mmHg).

After 48 hours of ICU stay for the correction of hypokalemia and metabolic acidosis, With improvement in potassium levels (K+ = 3.25mEq/L), we re-assessed patient's conditions, the muscle power and respiratory condition markedly improved, so weaning period is made and the patients is breathing normally without any difficult then was successfully extubated. She was then able to breathe normally support herself, move all the four limbs and vonate normally.

Plans for further testing including nerve conduction studies, spinal MRI, and CSF analysis were deferred and the patient transferred internal medicine ward to follow-up in further view of deranged electrolytes, after 2 day of stay in the ward the patients’ cause of hypokalemia is concluded as refractory hypokalemia and safely discharged.

## 3 Discussion

Acute neuromuscular paralysis is one of the common neurological emergencies of which Guillain Barre Syndrome (GBS) remains the most common cause.

In our patient, the clinical presentations and physical findings had similarity with Guillain Barre Syndrome, although more typically weakness progresses over several days in GBS rather than the rapid involvement of upper extremity muscles and respiratory muscles. Moreover, the absence of fever on admission speaks against GBS, as usually patients with GBS had fever for days or weeks prior to onset of the symptoms.

Furthermore,the current case denied a preceding history of respiratory tract infection or diarrhea and vomiting occurs in more than 50% of GBS patients and also showed a dramatic improvement of both neurologic and respiratory symptoms followed after correction potassium level. So it favors the rarely occurring manifestation of hypokalemia.

Hypokalemia is a well known cause of muscle weakness and mimics features with GBS which is many times overlooked or missed in differential diagnosis and extensive review of literature revealed only three case reports in which the patients had presented like GBS, but later the cause was found out to be hypokalemia [5].

Hypokalemia may result a wide variety of conditions due either to alteration in transcellular distribution of potassium, actual potassium depletion from renal or extra renal - mainly gastrointestinal – losses and decreased intake, and thyrotoxic periodic paralysis, while the most reported cases were associated in with familial periodic paralysis [1]. Although our patient had no history of thyrotoxicosis and familial periodic paralysis, and also patient denied exposure to barium, barium containing rat poisons, or renal disease, the actual cause of potassium depletion was due poor intake of the patient.

Acute hypokalaemic paralysis is a clinical condition presented with acute ascending, areflexic extremities,and weakness. In severe form of hypokalimia it can cause entire muscular paralysis, including respiratory, bulbar, and cranial muscles, as well as death through respiratory failure and arrhythmia [1].

In conclusion, the clinical presentations and physical findings of patient we seen were consistent to those of GBS and provisional diagnosis of GBS was made until routine electrolyte result was obtained with severe hypokalemia and diagnosis reconsidered to hypokalemia induced paralysis.

The recommendation from this case report is, every physician and intensivist working in emergency room, especially in resource limited setting whenever they are faced with a patient presenting to ER with acute ascending flaccid quadriparesis without sensory deficit, they should determine serum electrolyte for every such patient to consider severe hypokalemia as possible treatable differential diagnosis before unnecessary investigations and costly interventions. Hypokalemia should be aggressively controlled to ensure that patients recover quickly and good prognosis.

Further research is needed to measure the association between severe hypokalimia and ascending paralysis. Also, it is needed to evaluate the incidence and long-term outcome of ascending paralysis due to severe hypokalima.

## Ethics approval and consent to participate

Our institution does not require ethical approval for reporting individual case report.

## Funding

No funding was received.

## Author contributions

All authors performed substantial contributions to accession of data, or analysis, conception and design, and interpretation of data. Took part in drafting the article or revising it critically for important intellectual content and gave final approval of the version to be published.

## Registration of research studies

Name of the registry: **Not Applicable.**

Unique Identifying number or registration ID: **Not Applicable.**

Hyperlink to your specific registration (must be publicly accessible and will be checked): **Not Applicable.**

## Guarantor

As Corresponding Author, I confirm that the manuscript has been read and approved by all named authors.

## Consent to publish

Written informed consent was obtained from the patient for publication of this case report and accompanying images. A copy of the written consent is available for review by the Editor-in-Chief of this journal on request.

## Availability of data and materials

The data is available from the corresponding author and coauthors and can be accessed if requested.

## Declaration of competing interest

The author declares that they have no competing interests.
